# Experimental illumination of a forest: no effects of lights of different colours on the onset of the dawn chorus in songbirds

**DOI:** 10.1098/rsos.160638

**Published:** 2017-01-11

**Authors:** Arnaud Da Silva, Maaike de Jong, Roy H. A. van Grunsven, Marcel E. Visser, Bart Kempenaers, Kamiel Spoelstra

**Affiliations:** 1 Department of Behavioural Ecology and Evolutionary Genetics, Max Planck Institute for Ornithology, Seewiesen, Germany; 2 Department of Animal Ecology, Netherlands Institute of Ecology (NIOO-KNAW), Wageningen, The Netherlands; 3 Nature Conservation and Plant Ecology Group, Wageningen University, Wageningen, The Netherlands

**Keywords:** experimental night lighting, light colour, light-emitting diode, dawn singing, songbirds

## Abstract

Light pollution is increasing exponentially, but its impact on animal behaviour is still poorly understood. For songbirds, the most repeatable finding is that artificial night lighting leads to an earlier daily onset of dawn singing. Most of these studies are, however, correlational and cannot entirely dissociate effects of light pollution from other effects of urbanization. In addition, there are no studies in which the effects of different light colours on singing have been tested. Here, we investigated whether the timing of dawn singing in wild songbirds is influenced by artificial light using an experimental set-up with conventional street lights. We illuminated eight previously dark forest edges with white, green, red or no light, and recorded daily onset of dawn singing during the breeding season. Based on earlier work, we predicted that onset of singing would be earlier in the lighted treatments, with the strongest effects in the early-singing species. However, we found no significant effect of the experimental night lighting (of any colour) in the 14 species for which we obtained sufficient data. Confounding effects of urbanization in previous studies may explain these results, but we also suggest that the experimental night lighting may not have been strong enough to have an effect on singing.

## 1. Introduction

Natural patterns of day and night have occurred on Earth for several billion years until artificial light started to pervade the environment [1]. Coined ‘light pollution’, this recent increase in the number of artificial light sources is increasingly perceived as a potential threat for wildlife [2,3]. Artificial illumination of oil platforms and lighthouses disorients and kills millions of nocturnally migrating birds each year [4]. Light pollution also interferes with photoperiod, thereby disrupting the temporal timing of animal behaviour, both on a seasonal and a daily basis [5–8].

Songbirds use the rise in natural light levels at dawn as a cue to initiate singing, possibly because low light intensities reduce foraging success and thus favour other behaviours [9,10]. Dawn song is used by male songbirds to announce territory ownership and to attract or guard mates [10,11]. In the great tit *Parus major*, mate fertility and her time of emergence from the nest seem to determine the duration of dawn singing [12]. The onset of dawn singing is also suggested to be one reliable indicator of male quality or age, with higher reproductive success for the earliest initiators of dawn song [13–15].

Several studies indicate that light pollution causes males of several songbird species in temperate latitudes to sing earlier at dawn [16–19], which may ultimately impact reproductive success [17]. However, all of these studies are correlational (but see also [19]), i.e. the link between early singing and artificial light may have been confounded by other anthropogenic factors or by differences between urban and rural birds. More experimental work is thus needed. Secondly, previous work has only investigated the effect of conventional (white) lighting on timing of dawn song, and the effect of different light colours is still unknown. Daily rhythms may be affected by spectral composition because birds possess three photoreceptive sites involved in circadian rhythmicity (the retina, the pineal gland and the hypothalamus) with different spectral sensitivities [20,21]. Because the use of solid-state lighting such as outdoor LED lights with customizable colour composition is steadily increasing [22], knowledge about the impact of different LED colours on timing of singing may be useful in order to mitigate light effects.

In our experiment, we controlled for potentially confounding effects of urbanization by experimentally illuminating forests in dark natural areas with white, green and red LED lights (and a dark control). We recorded daily dawn chorus of all songbird species singing near each light-treated area and predicted that the onset of dawn song would be earlier in the light-treated compared with the control areas. The white and green treatments may have the strongest effect, because photoreceptors are more sensitive to shorter wavelengths [20], and because red light may be perceived by birds as less intense than other colours [21]. On the other hand, red light may have a stronger effect than green light because it more easily penetrates the skull to reach the hypothalamus [21]. We expected stronger light effects for the early-singing species, which are in general more sensitive to natural [23,24] and artificial [18,19] light variation.

## 2. Material and methods

### 2.1. Experimental set-up

We illuminated eight previously dark natural forest edges in The Netherlands with conventional street lights from sunset until sunrise. Forests were far from urban areas and thus not exposed to potentially confounding factors such as noise pollution, increased temperatures and bird feeders. Each site had four 100 m-long transects with five lamp posts (height 4 m) perpendicular to the forest edge, with two light posts in the forest, one at the forest edge and two in the open field (see fig. 2 in study [25] for a schematic overview of the set-up). The distance between transects varied between 88 and 386 m (average ± s.e.: 204 ± 17 m). Each transect produced either commercially available (Philips Fortimo) white, (Clearsky) green, or (Clearfield) red lights, or no light (dark control). All lamps emitted full spectrum light with negligible UV emission, but green lamps had an increased blue and a reduced red emission, whereas this was the opposite for red lamps. Mean light intensity underneath the light posts was 7.4 ± 0.3 lux at ground level (white light 10.1 lux, green light 7.0 lux, red light 5.7 lux). Electronic supplementary material, fig. S1, in study [26] shows how light intensities at the nest-box level decrease with distance from the nearest lamp post, for all light colours. Lighting started in 2012, except for one site, which was illuminated only from 26 April 2013 onwards. Breeding was monitored in 2013 in great tits (*N* = 96 broods in 2013; mean lay date = 1 May), pied flycatchers *Ficedula hypoleuca* (*N* = 47; 9 May) and blue tits *Cyanistes caeruleus* (*N* = 9; 1 May), that bred in nest-boxes at each transect. Note that 2013 was a cold year with a late breeding season.

### 2.2. Data collection and extraction

Each day from late February to May 2013, we recorded the dawn chorus from 3.5 h before until 1 h after local sunrise, using Song Meter SM2+® recorders (Wildlife Acoustics, Concord, MA, USA), which we attached in each transect to the second-to-last lamp post in the forest (height 3 m, *N* = 32 recorders). Next, we collected and blindly analysed the resulting sound files using Song Scope® 4.1.1 (Wildlife Acoustics). We only extracted the data collected between 15 April and 15 May (27 April–15 May for the late-lighted site; 4248 h of recording, 944 recorder days in total), which corresponded to the peak intensity of singing for a majority of species (based on field observations and on the analysis of a subsample of sound files outside this period). We noted on each day, for each transect, the time of the first song for each passerine species if the same species produced two more strophes within 5 min, and if these three songs were louder than −50 dB. This threshold was initially selected by comparing sonograms from adjacent transects, and it generally included songs occurring within a 50–80 m range around each recorder.

### 2.3. Statistical analyses

**Figure 1.**
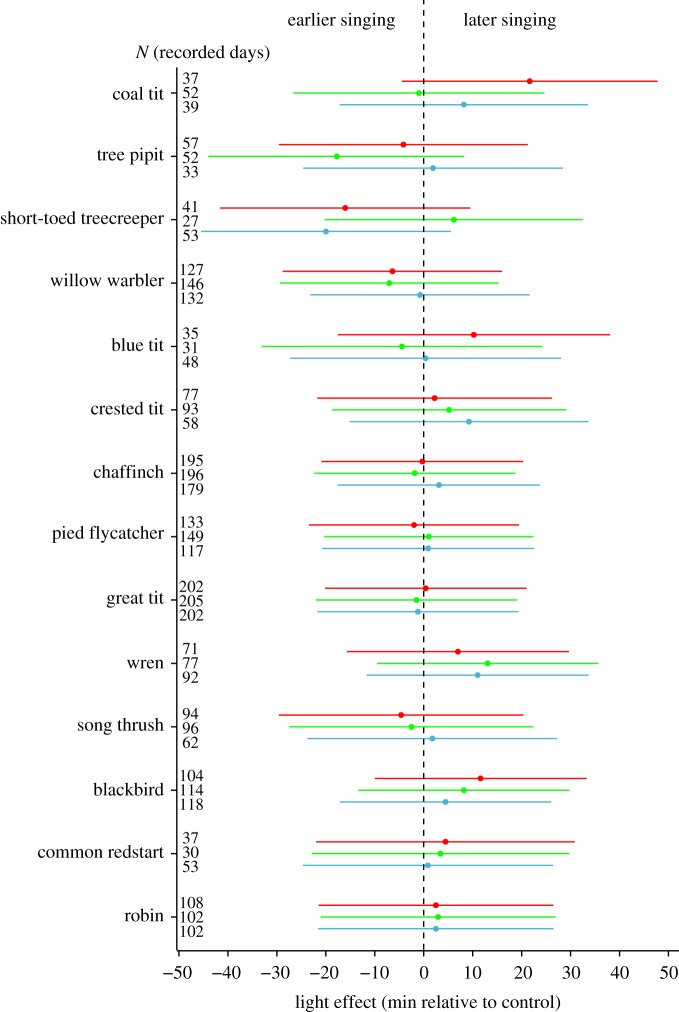
Estimates (±95% confidence interval) of the effects of the light treatment (blue for the white (light) treatment, green for the green (light) treatment and red for the red (light) treatment) on the onset of dawn singing relative to the onset in the control for 14 passerine species (see Material and methods). Light effects are significant when the confidence interval does not overlap zero. Species are ordered from earliest (bottom) to latest (top) singers (according to mean onset in the control). Sample sizes (number of recorded days) are given for each treatment and species next to each estimate.

We analysed data from 14 species that were detected on at least 62 recorder days in the control treatment (see figure 1 and table 1 for sample sizes). All statistical analyses were performed with the R 3.1.2 software [27], using linear mixed-effect models (LMMs fit by ML, R-package *nlme* [28]), with ‘species’ nested in ‘site’ as a random intercept, and ‘date’ as a random slope (to control for temporal autocorrelation). Note that ‘site’ has seven levels here because we merged two sites that were spatially intermingled. Two other sites were adjacent but not intermingled, and the four last sites were located in four different regions. In those models, we used the ‘onset of dawn song relative to control’ as a response variable. This is calculated for each species, at each site and on each day, as the difference (in min) between the onset of dawn song in each light treatment (red/green/white) and the onset of dawn song in the dark control of the same site on the same day. Testing whether this response variable differs from zero is an alternative to testing the overall difference in onset of singing between the four treatment groups (which gives qualitatively similar results), but is better because it allows for direct day-to-day comparisons within each site, thereby controlling for potentially confounding effects of season, weather or other local conditions that may influence timing of singing. First, we tested for a general treatment effect using ‘treatment’ (red/green/white) as a fixed effect. Second, we tested for treatment effects within each species by using ‘treatment’ in interaction with ‘species’ as a fixed effect (42 levels). In the *post hoc* tests of the two models, we corrected for multiple testing using the *multcomp* R-package (single-step method) [29], and we calculated the conditional *R*^2^ to assess their predictive quality [30]. Note that correction for the large number of comparisons may lead to overly conservative *p*-values; hence, the focus should be on effect sizes and confidence intervals. Finally, we tested for each light colour whether the light effect for a particular species depended on the natural song onset of that species using Pearson's correlations between the ‘mean onset of dawn song relative to control’ for a given species and the species ‘mean onset of dawn song in the control’.

**Table 1.** Results of *post hoc* tests from linear mixed models (LMMs) showing the effects of the three light treatments on the onset of dawn singing relative to the dark control treatment in 14 passerine bird species. Positive estimates indicate later singing under the light treatment compared with the control, negative values indicate earlier singing. Species are ordered according to the mean onset in the control treatment. Sample sizes (number of recorded days) are given in brackets for each species. Next to the treatment for each species, we also provide the number of sites at which the species was heard on 5 or more days (5 days was chosen as an arbitrary cut-off to exclude rare singing events), to show the estimated sample size for each species.

treatment	estimates^a^	s.e.^b^	*z*-value	*p*-value^c^
European robin *Erithacus rubecula* (*N* = 312)				
white (4)	2.5	7.7	0.3	1.0
green (4)	2.9	7.7	0.4	1.0
red (4)	2.5	7.7	0.3	1.0
common redstart *Phoenicurus phoenicurus* (*N* = 120)				
white (3)	0.9	8.1	0.1	1.0
green (3)	3.4	8.5	0.4	1.0
red (3)	4.4	8.5	0.5	1.0
common blackbird *Turdus merula* (*N* = 336)				
white (5)	4.4	6.9	0.6	1.0
green (5)	8.2	6.9	1.2	1.0
red (5)	11.6	7.0	1.7	0.9
song thrush *Turdus philomelos* (*N* = 252)				
white (4)	1.8	8.2	0.2	1.0
green (5)	−2.5	8.0	−0.3	1.0
red (5)	−4.6	8.0	−0.6	1.0
Eurasian wren *Troglodytes troglodytes* (*N* = 240)				
white (5)	11.0	7.3	1.5	1.0
green (5)	13.1	7.3	1.8	0.8
red (5)	7.0	7.3	1.0	1.0
great tit *Parus major* (*N* = 609)				
white (7)	−1.2	6.6	−0.2	1.0
green (7)	−1.5	6.6	−0.2	1.0
red (7)	0.4	6.6	0.1	1.0
pied flycatcher *Ficedula hypoleuca* (*N* = 399)				
white (6)	0.9	7.0	0.1	1.0
green (6)	1.1	6.9	0.2	1.0
red (6)	−2.0	6.9	−0.3	1.0
common chaffinch *Fringilla coelebs* (*N* = 570)				
white (7)	3.1	6.6	0.5	1.0
green (7)	−1.9	6.6	−0.3	1.0
red (7)	−0.3	6.6	−0.05	1.0
crested tit *Lophophanes cristatus* (*N* = 228)				
white (4)	9.3	7.8	1.2	1.0
green (4)	5.2	7.7	0.7	1.0
red (4)	2.2	7.7	0.3	1.0
blue tit *Cyanistes caeruleus* (*N* = 114)				
white (3)	0.4	8.9	0.05	1.0
green (2)	−4.4	9.2	−0.5	1.0
red (2)	10.3	8.9	1.1	1.0
willow warbler *Phylloscopus trochilus* (*N* = 405)				
white (6)	−0.8	7.2	−0.1	1.0
green (6)	−7.0	7.2	−1.0	1.0
red (6)	−6.4	7.2	−0.9	1.0
short-toed treecreeper *Certhia brachydactyla* (*N* = 121)				
white (3)	−20.0	8.2	−2.4	0.3
green (2)	6.1	8.5	0.7	1.0
red (3)	−16.0	8.2	−2.0	0.7
tree pipit *Anthus trivialis* (*N* = 142)				
white (3)	1.9	8.5	0.2	1.0
green (4)	−17.7	8.4	−2.1	0.6
red (3)	−4.1	8.2	−0.5	1.0
coal tit *Periparus ater* (*N* = 128)				
white (3)	8.2	8.2	1.0	1.0
green (2)	−1.0	8.3	−0.1	1.0
red (2)	21.6	8.4	2.6	0.2

^a^Minutes relative to the dark control (negative values imply earlier singing under the light treatment).

^b^Standard error.

^c^*p*-Values after correction for multiple testing (42 comparisons).

## 3. Results

We found no overall effect of the light treatment on the daily onset of dawn song relative to the dark control (white: 2.1 min ± 2.1, *z* = 1.0, *p* = 0.5; green: 0.6 ± 2.1, *z* = 0.3, *p* = 0.9; red: 1.5 ± 2.1, *z* = 0.7, *p* = 0.6; model $R_{c}^{2}=0.5$). We also did not find any effect of the light treatment on the daily onset of dawn song relative to the dark control in any of the 14 species (figure 1 and table 1; $R_{c}^{2}=0.5$). Contrary to our prediction, among species the mean onset of dawn song relative to the dark control correlated negatively with the mean onset in the control (i.e. light effects generally increased from early to late singers; figure 1), but this was not significant (white: *r*_12_ = −0.3, *p* = 0.3; green: *r*_12_ = −0.4, *p* = 0.1; red: *r*_12_ = −0.5, *p* = 0.1).

## 4. Discussion

We found no experimental evidence that artificial night lighting from conventional white, green or red street lights advanced the daily onset of dawn singing in wild forest passerines singing near the light posts. Unexpectedly, experimental night lighting did not affect naturally early singers more than late singers.

For some species, the absence of effects may be explained by their ecology. For example, those species which generally hold large or open territories (e.g. thrushes or redstarts), or which show low local densities (e.g. coal tits), may not have been much affected by the light because they were less tied to any transects. Some species may also have avoided settlement in the lighted territories, as shown in a previous study using the same set-up [25]. By contrast, light effects should have been present in those species that bred in the local nest-boxes, i.e. the closest to the lamp posts (i.e. great tits, blue tits and pied flycatchers), but this was not the case or the effects may have been too small to be detected. Finally, some species may have shown earlier singing under the artificial light treatment outside the month-long study period but the analysis of a larger sample of sound files for the white light and control-treated areas indicates that this was not the case.

We did not find any evidence for an advancing effect of the experimental light in those species which showed clear and consistent advances in singing [18,19] or activity [30] onset in night-lighted habitats in previous studies (i.e. great tits, blue tits, song thrushes, blackbirds and robins). Moreover, for these species, the mean onset of dawn song in our experimental light set-up was comparable to the mean natural onset of singing of these species in the unlighted control sites of study [18] (this study versus [18]: robin: −59.3 min (relative to sunrise) ± 5.5 versus −57.7 ± 19.1; blackbird: −49.2 ± 7.1 versus −53.2 ± 17.3; song thrush: −50.1 ± 8.2 versus −49.1 ± 14.8; great tit: −34.3 ± 5.3 versus −31.4 ± 25.6; blue tit: −18.8 ± 14.6 versus −16.2 ± 25.3; chaffinch: −17.3 ± 7.7 versus −9.1 ± 18.0). Thus, our results suggest that the earlier singing previously observed in lighted habitats may have been influenced by (intrinsic or learned) behavioural or physiological differences between urban and forest birds [31,32], or by other anthropogenic factors covarying with artificial light such as noise pollution [33,34].

However, a recent experiment suggests that artificial night lighting alone is able to drive early singing in birds breeding in natural forests such as robins [19]. Because in the latter experiment strong light intensities were used and because the effects of light pollution on timing of singing or activity have been shown to increase with light intensity [6,16,18,35], it is more probable that detectable light effects on onset of dawn song only occur at high light intensities. Indeed, the ground light intensity used in our experiment falls within the lower part of the intensity range of the sites in [18] (sites with weak effects: [1.5–7.0 lux at ground level] versus sites with strong effects [21.0–30.1 lux]). Moreover, light intensity decreased with distance from the lamp post (fig. S1 in study [26]), partly due to vegetation cover, so birds singing at a range of 40–80 m from the lights may have been exposed to very low light intensities (less than 0.01 lux), depending on their song perch. This highlights that our experiment may have been limited in terms of illumination even though we had expected that onset of singing of birds singing further away from the lights could still be influenced via direct or indirect effects of low light intensities on avian activity rhythms [6].

The street lights used in this experiment have conventional light intensities and are commonly used for countryside road lighting. Our results thus suggest that such street lights in such a configuration have a minimal impact on timing of dawn singing and may therefore be relatively suitable for use in natural environments. Contrary to our predictions, there was no differential impact on timing of dawn singing between the light colours. However, given that even the white light treatment had no effect here, and that our experimental birds may have been exposed to low light levels at night, more experimental research is needed, either in the field or in laboratory settings.

Finally, the absence of an advancing effect on the timing of dawn singing during the breeding season does not exclude longer-term effects of exposure to artificial night lighting on singing behaviour, for example due to direct processes such as learning or selective settlement of early birds near the light, or due to indirect processes such as the influence of altered prey abundance on reproductive behaviour. We also cannot exclude that artificial night lighting affects other traits such as hormonal levels and physiology [35–39], sleep [40,41] and lay date [17,42].

## Data accessibility

Data are available at the Dryad Digital Repository http://dx.doi.org/10.5061/dryad.437t2 [43].
